# Higher Decorin Levels in Bone Marrow Plasma Are Associated with Superior Treatment Response to Novel Agent-Based Induction in Patients with Newly Diagnosed Myeloma - A Retrospective Study

**DOI:** 10.1371/journal.pone.0137552

**Published:** 2015-09-17

**Authors:** Shang-Yi Huang, Hsiu-Hsia Lin, Ming Yao, Jih-Luh Tang, Shang-Ju Wu, Hsin-An Hou, Wen-Chien Chou, Sheng-Chieh Chou, Szu-Chun Hsu, Bor-Sheng Ko, Hsiao-Yun Lu, Woei Tsay, Hwei-Fang Tien

**Affiliations:** 1 Department of Internal Medicine, National Taiwan University, Medical College and Hospital, Taipei, Taiwan; 2 Department of Laboratory Medicine, National Taiwan University, Medical College and Hospital, Taipei, Taiwan; Imperial College London, UNITED KINGDOM

## Abstract

The growth of myeloma cells depends on bone marrow (BM) stroma consisting of stromal cells, secreted cytokines and the extracellular matrix (ECM). Decorin, a small leucine-rich proteoglycan in the ECM, is a signaling ligand and native anti-tumor agent. However, the role of decorin in patients with myeloma is not clear. We evaluated the correlation between the decorin levels measured by enzyme-linked immunosorbent assay in BM plasma from 121 patients with newly diagnosed myeloma based on their clinical features and treatment response. The median decorin levels in the patients and the normal control group were 12.31 ng/mL [standard deviation (SD), 7.50 ng/mL; range, 2.45 to 44.46 ng/mL] and 10.31 ng/mL (SD, 2.42 ng/mL; range, 4.85–15.14 ng/mL), respectively (*P* < 0.001). Using 15.15 ng/mL as a cut-off, 46 patients (38%) exhibited higher decorin levels (H-DCN), whereas the other patients exhibited normal to lower decorin levels (NL-DCN). Except for the median age, which was significantly younger in the H-DCN than in the NL-DCN group (60.6±14.0 vs. 65.8±12.2 years, respectively; *P* = 0.034), there were no differences between the two groups. However, in 79 patients who had received novel agent-based induction, the overall response rate was significantly better in the H-DCN than in the NL-DCN (97 vs. 63%, respectively; *P* < 0.001), as was the depth of responses (*P* = 0.008), which were not observed in those who had received chemotherapeutic agents alone. Progression-free survival (PFS) was significantly longer in H-DCN than NL-DCN (not reached vs. 19.5 mo, respectively; *P* = 0.0003). Multivariate analyses indicated that H-DCN, as a significantly independent factor, was associated with better treatment response (odds ratio, 20.014; 95% CI, 2.187–183.150; *P* = 0.008) and longer PFS (hazard ratio, 0.135; 95% CI, 0.051–0.361; *P* < 0.001). These findings disclose the potential role of decorin in myeloma and provide a basis for further study on possible synergistic anti-myeloma effects between decorin and the novel agents that target BM stroma.

## Introduction

Multiple myeloma (MM) is monoclonal plasma cell proliferation in the bone marrow (BM) [1]. Over the past decade, considerable progress has been achieved in the available treatment options for MM with several novel agents including proteasome inhibitors (PIs) (e.g., bortezomib [BTZ] and carfilzomib [CFZ]) and immunomodulatory drugs (IMiDs) (e.g., thalidomide [THA], lenalidomide, and pomalidomide), which have improved the survival of patients with MM [1, 2]. A major advantage of using these novel agents in treating MM is that they not only induce apoptosis of myeloma cells (MCs) but also interrupt cross interactions between the MCs and the adjacent BM stroma [3, 4]. Typically, the growth of MCs strongly depends on the BM stroma, which primarily comprises stromal cells, secreted cytokines, and the extracellular matrix (ECM) [5]. Although the stromal cells and secreted cytokines are known to regulate growth, drug resistance, angiogenesis, and the extramedullary expansion of MCs [4, 5], the role of the ECM remains unclear.

The ECM is comprised of collagens and non-collagens [6]. Small leucine-rich repeat proteoglycans (SLRPs) form the major non-collagen component of the ECM [6, 7]. Decorin belongs to SLRPs class I and is involved in the regulation of collagen fibrillogenesis [7, 8]. Mutations in decorin lead to connective tissue disorders such as congenital stromal corneal dystrophy [9, 10]. In addition to such structural components, subsequent studies have revealed that decorin may sequester multiple growth factors, such as transforming growth factor-β (TGF-β) [11], and may bind to several receptor tyrosine kinases, including epidermal growth factor receptor (EGFR), insulin-like growth factor-I receptor (IGF-IR), and hepatocyte growth factor (HGF) receptor (Met), etc. [8, 11]. The decorin–EGFR interaction triggers a signal cascade that activates the mitogen-activated protein kinases, up-regulation of p21^WAF1/CIP1^, and, eventually, growth suppression [8, 11]. Several studies on decorin-deficient mice have shown a tendency to develop intestinal tumors and enhanced hepatic carcinogenesis [12, 13]. Furthermore, these studies have shown a combined genetic ablation of decorin- and p53-induced T-cell lymphoma [14]. More recently, decorin was implicated in modulating inflammatory responses with strong antitumor effects [15]. Therefore, decorin is now being reconsidered as a novel and native signaling ligand rather than a structural protein alone, that contributes to numerous pathophysiological processes, including inhibition of tumor growth, metastasis, and angiogenesis [7, 8].

Unlike the extensive studies evaluating the roles of decorin in solid cancers, the role of decorin in hematological malignancies has not been completely explored. Until now, only a few studies have been conducted in MM that showed the downregulation of decorin expression in the BM plasma of patients with MM compared to healthy donors [16], which was even lower in MM patients with lytic bone lesions [17]. The decorin that is expressed in BM mesenchymal stem cells (BMMSCs) and osteoblasts (OBs) can directly and indirectly suppress the growth of MCs [18, 19]. However, the relationship between decorin levels and clinical features in patients with MM remains unclear. Thus, the present study evaluated the correlation between decorin levels in the BM plasma and clinical features and treatment outcome in 121 patients with newly diagnosed multiple myeloma (NDMM) to explore the possible roles of decorin in MM.

## Materials and Methods

### Patients and bone marrow plasma

Between July 1998 and August 2014, 121 patients with NDMM were enrolled. The diagnosis of MM was confirmed according to the International Myeloma Working Group (IMWG) criteria [20]. Upon diagnosis, BM plasma samples (10–20 mL) were collected and processed as previously described [21] using anticoagulant heparin and stored at −80°C until analysis. In addition, BM plasma samples were collected from 22 healthy BM donors comprised of 15 men and 7 women with a median age of 46 years (range, 20–68 y) and used as normal controls. This study and consent procedure was approved by the National Taiwan University Hospital Research Ethics Committee (NTUHREC: 201212114RINC), and written informed consent was obtained from all study participants in accordance with the Declaration of Helsinki and kept in their medical records.

### Enzyme-linked immunosorbent assay (ELISA) for decorin

The decorin levels in the archived BM plasma samples were measured using DuoSet enzyme-linked immunosorbent assay (ELISA) kits (R&D systems, Minneapolis, MN, USA), according to the manufacturer’s instructions. In brief, 96-well plates were coated with 2 μg/mL of capture antibodies per well and incubated overnight at room temperature (RT). In addition, 1% bovine serum albumin (Bionovas, Toronto, Canada) in PBS (200 μL/well) was added to block nonspecific binding. Then, 100 μL of plasma sample (diluted if indicated) was added to each well and incubated overnight at 4°C. A detection antibody (200 μL) was added and incubated at RT for 2 hours. Subsequently, the wells were incubated with a streptavidin-HRP conjugated antibody for 20 minutes followed by 100 μL of substrate solutions for 20 minutes, and then with a stopping solution. The wells were then placed on an ELISA plate reader (Perkin Elmer, CA, USA), and the absorbance at 450 nm of each well was recorded. Serial dilutions (0–800 ng/μL) of purified decorin protein were used as standards in each experiment. The detection limits of decorin in the plasma ranged between 31.3 and 2000 pg/mL. All experiments were repeated at least 3 times, and the mean decorin levels (ng/mL) were calculated and presented.

### Induction regimen, treatment response and outcome

The individual induction regimen was prescribed based on a patient’s condition and their doctor’s discretion. The uniform treatment schedules were as follows: conventional chemotherapeutic agents, VAD (28-d cycle: vincristine 0.4 mg/m^2^, continuous intravenous infusion [CIVF], days 1–4; doxorubicin, 9 mg/m^2^, CIVF, days 1–4; oral dexamethasone 40 mg/d, days 1–4 and 8–11) and MP (28-d cycle; oral melphalan 9 mg/m^2^ and prednisolone 60 mg/m^2^/d on days 1–4); and novel agent-based regimens, BTD (21-d cycle: BTZ 1.3 mg/m^2^ [sc or iv] on days 1, 4, 8, and 11; THA 100–200 mg/d; oral dexamethasone 20–40 mg/d on days 1–4;), and BTD+Cy (21-d cycle; BTD + oral cyclophosphamide 100 mg/d on days 1–4). High dose melphalan (200 mg/m^2^) was used for the conditioning of autologous stem cell transplant (HDC/AuSCT) and 50–100 mg/d THA was provided for post AuSCT maintenance. The treatment response, progression-free survival (PFS) of the frontline therapy, and the overall survival (OS) in each patient were reevaluated based on the IMWG criteria [22].

### Statistics

Chi-square or Fisher’s exact tests were used for intergroup comparisons of the discrete variables. A two-sample *t* test was used for intergroup comparisons of the means. Pearson’s correlation test was used for the correlation between continuous variables. Kaplan–Meier survival curves were constructed to estimate PFS and OS, and the intergroup differences were compared using a log-rank test. In the analyses, identified salient variables for the clinical and laboratory data were categorized as described previously [23] and listed as follows: age ≥ 60 years; stage ≥ Durie-Salmon Stage III; stage ≥ International Staging System III; non-IgG isotype; Hb < 10 gm/dL; WBC < 4.0 × 10^9^/L; PLA < 1.5 × 10^11^/L; LDH ≥ upper limit of normal range (ULN); ALP ≥ ULN; Ca ≥ 2.4 μmol/L; Cr ≥ 2.0 mg/dL; and C-reactive protein (CRP) ≥ ULN (0.8 mg/dL). High risk cytogenetics denote any clonal changes detected by a conventional G-banding (CG) technique, and/or t (4;14), t (14;16) and del (17p) detected by fluorescence in-situ hybridization (FISH), performed as previously described [23]. Factors that provided statistically significant predictive power in the univariate analysis were further subjected to multivariate regression analysis of the linear, logistic, or Cox type. At Cox regression analysis, age was considered as a continuous covariate. Regarding the treatment response, the best cut-off value of decorin was selected by the Youden’s index using the receiver operating characteristic (ROC) curve, and the area under the ROC curve (AUC) was calculated. All directional *P* values were 2-tailed, and *P* ≤ 0.05 was considered significant for all tests. All analyses were performed using the SPSS Version 19.0 software (Chicago, IL, USA).

## Results

### Patients and induction treatment

Table 1 summarizes the clinical features of the 121 patients at diagnosis. Of the 121 patients, 79 (65%) had received novel agent-based induction regimens, including BTD (53 patients) and BTD+Cy (26 patients), whereas the other patients (42 patients, 35%) had received conventional chemotherapeutic agent-based regimens, including VAD (16 patients) and MP (26 patients). S1 Fig presents the patient disposition and treatment duration of these induction regimens. Table 2 summarizes the treatment responses to these various induction regimens. Notably, there were no significant differences in treatment response between the BTD and BTD+Cy regimens.

**Table 1:** Clinical characteristics of the 121 patients with NDMM and the comparison between those who had high (H-) and normal/low (NL-) decorin level in their BM plasma.

Patients	All	H-DCN	NL-DCN	
N	121	46	75	*P*-value
Sex (M/F)	68/53	24/22	44/31	0.572
Age (yrs)*	63.8±13.1	60.6±14.0	65.8±12.2	0.034
DSS [N (%)]				0.265
I/II	55 (45)	24 (52)	31 (41)	
IIIa/b	66 (55)	22 (48)	44 (59)	
ISS [N (%)]				0.850
I/II	64 (53)	23 (50)	41 (56)	
III	57 (47)	23 (50)	34 (44)	
Isotype [N (%)]				0.299^#^
IgG	62 (51)	24 (52)	38 (51)	
IgA	28 (23)	7 (15)	21 (28)	
IgD	4 (3)	2 (4)	2 (3)	
Light-chain	26 (22)	13 (29)	13 (17)	
IgM	1 (1)	0 (0)	1 (1)	
Kappa:Lambda ratio	1.2:1	0.8:1	1.6:1	0.131
Hemoglobin (gm/dL)*	9.7±2.8	9.9±3.1	9.6±2.6	0.595
White blood cell (x10^9^/L)*	7.3±4.4	7.5±4.6	7.2±4.3	0.662
Platelet (x10^11^/L)*	2.1±1.0	2.3±1.1	2.0±0.9	0.130
Creatinine (mg/dL)*	2.1±2.3	2.0±2.2	2.2±2.3	0.615
Calcium (μmol/L)*	2.3±0.3	2.3±0.4	2.3±0.3	0.825
LDH (IU/L)*	341±229	338±211	343±240	0.908
ALP (IU/L)*	182±361	154±124	199±450	0.517
CRP (mg/dL)*	1.9±3.3	1.6±3.0	2.0±3.5	0.522
Albumin (gm/dL)*	3.5±0.8	3.6±0.9	3.5±0.8	0.646
β_2_M (mg/L)*	10.6±13.2	10.8±13.9	10.4±12.8	0.883
Plasma cell in BM (%)*	58.3±27.9	60.3±26.9	57.1±28.6	0.561
High risk CAs [N (%)]	35 (29)	14 (30)	21 (28)	0.837
CAs detected by CG	22 (18)	10 (22)	12 (16)	0.471
FISH_t(4;14)	13 (11)	5 (11)	8 (11)	1.000^#^
FISH_t(14;16)	1 (1)	0 (0)	1 (1)	1.000^#^
FISH_del 17p	8 (7)	3 (7)	5 (7)	1.000^#^
EMD [N (%)]	29 (24)	10 (22)	19 (25)	0.670
Amyloidosis [N (%)]	12 (10)	7 (15)	5 (7)	0.208^#^
Bone lesions [N (%)]				0.568
Lytic only	31 (26)	12 (26)	19 (25)	
With any fracture	54 (44)	18 (39)	36 (48)	0.354
Degeneration only	36 (30)	16 (35)	20 (27)	

*. mean±SD

^#^. Fisher’s exact test

Abbreviations: ALP, alkaline phosphatase; BM, bone marrow; CAs, cytogenetic abnormalities; CG, conventional G-banding; CRP, C-reactive protein; DCN, decorin; DSS, Durie-Salmon staging; EMD, extramedullary disease; F, female; ISS, International staging system; LDH, lactate dehydrogenase; M, male; NDMM, newly diagnosed multiple myeloma; β_2_M, beta_2_-microglobulin

**Table 2:** The treatment response of various induction regimens for the 121 patients with NDMM.

Induction (N)	Chemotherapeutic agents-based (42)			Novel agents-based (79)		
Regimen (N)	VAD (16)	MP (26)	*P*-value	BTD (53)	BTD+Cy (26)	*P*-value
		N (%)			N (%)	
CR*	0 (0)	0 (0)	0.144^#^	9 (17)	7 (27)	0.602^#^
VGPR	3 (19)	1 (4)		13 (25)	7 (27)	
PR	7 (44)	8 (31)		18 (34)	7 (27)	
SD	5 (31)	16 (62)		9 (17)	5 (19)	
PD	1 (6)	1 (3)		4 (7)	0 (0)	
ORR (> = PR)	10 (63)	9 (35)	0.113	40 (76)	17 (81)	0.777

*. Denote immunofixation negative complete response

^#^. Fisher’s exact test

Abbreviations: CR, complete response; NDMM, newly diagnosed multiple myeloma; VGPR, very good partial response; PR, partial response; SD, stable disease; PD, progressive disease; ORR, overall response rate

### Decorin

**Fig 1:**
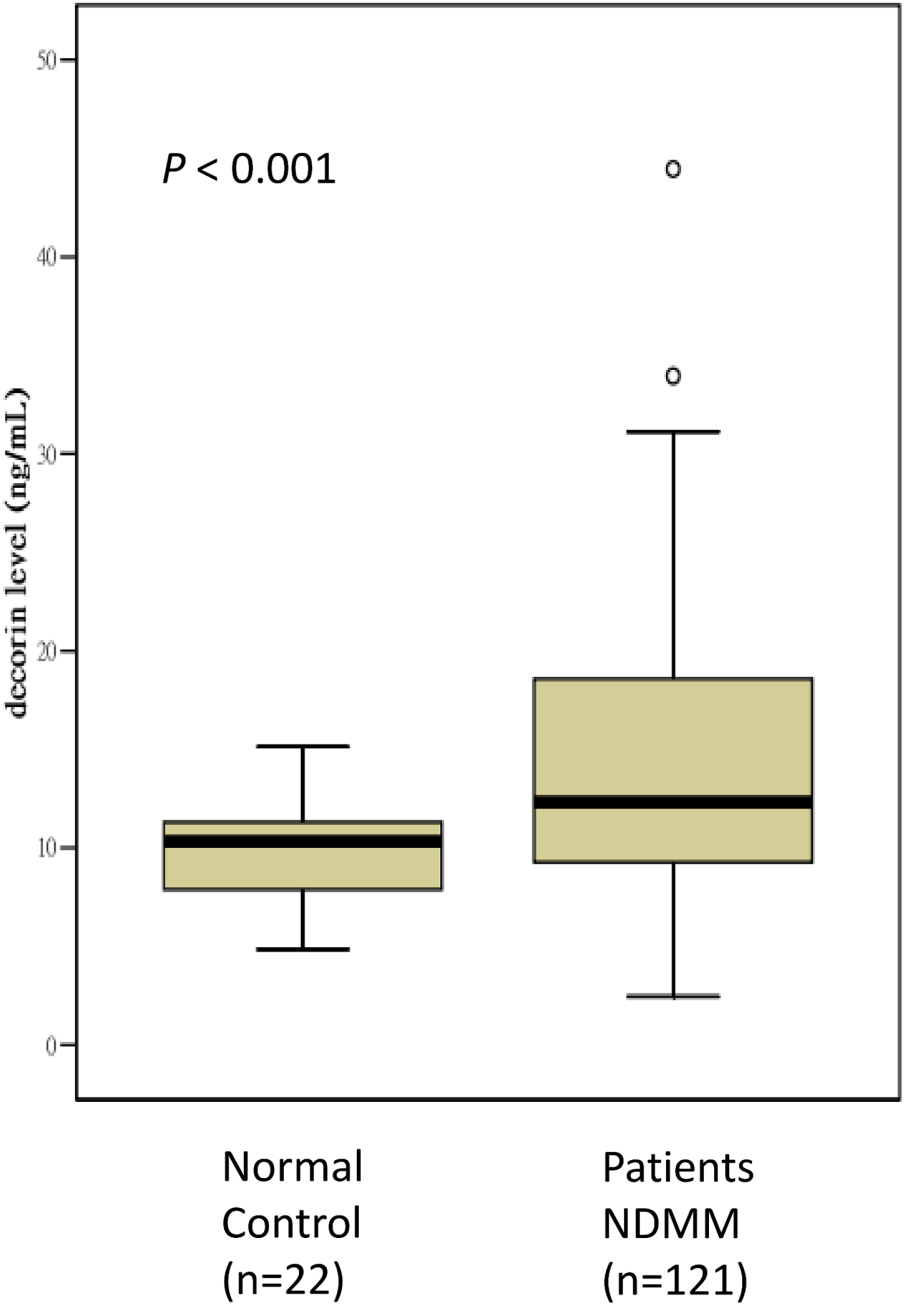
The distribution of decorin levels between the normal control patients and the patients with NDMM.

The median decorin level in the BM plasma of the 121 patients was 12.31 ng/mL with a standard deviation (SD) of 7.50 ng/mL, interquartile range (IQR) from 9.25 to 19.01 ng/mL, and range from 2.45 to 44.46 ng/mL. One-third of these samples (n = 41) were collected before 2010, and the median decorin level was not different between the samples collected before and after 2010 (12.17 ng/mL with IQR: 9.85 to 18.99 ng/mL vs. 12.77 ng/mL with IQR: 8.64 to 19.23 ng/mL, respectively; *P* = 0.946). The median decorin level in the control group was 10.31 ng/mL with SD of 2.42 ng/mL, IQR from 7.86 to 11.32 ng/mL, and range from 4.85 to 15.14 ng/mL. All plasma samples were analyzed at least twice, and the mean ± SD intra-assay coefficient of variability (CV) was 4.0±3.3%. There was a significantly positive correlation between decorin level and age in the normal control (Pearson’s correlation, 0.506; *P* = 0.016). The distribution of decorin levels between the patient and control groups is shown in Fig 1, and the difference between the two groups was statistically significant (*P* < 0.001).

### Clinical characteristics

Using the level just above the upper limit of decorin levels from the control group (15.15 ng/mL) as a cut-off, 46 (38%) of the 121 patients exhibited high decorin levels (higher decorin levels than the cut-off [H-DCN] group), whereas the other 75 patients (62%) exhibited normal to low decorin (lower decorin levels than the cut-off [NL-DCN] group). Table 1 and S1 Table present a comparison between the clinical characteristics and induction regimens of the H- and NL-DCN groups, respectively. Except for the median age that was significantly younger in the H-DCN group than in the NL-DCN group (60.6±14.0 vs. 65.8±12.2 years, respectively; *P* = 0.034), there were no differences in clinical features or induction regimens between the H- and NL-DCN groups.

### Treatment response

**Fig 2:**
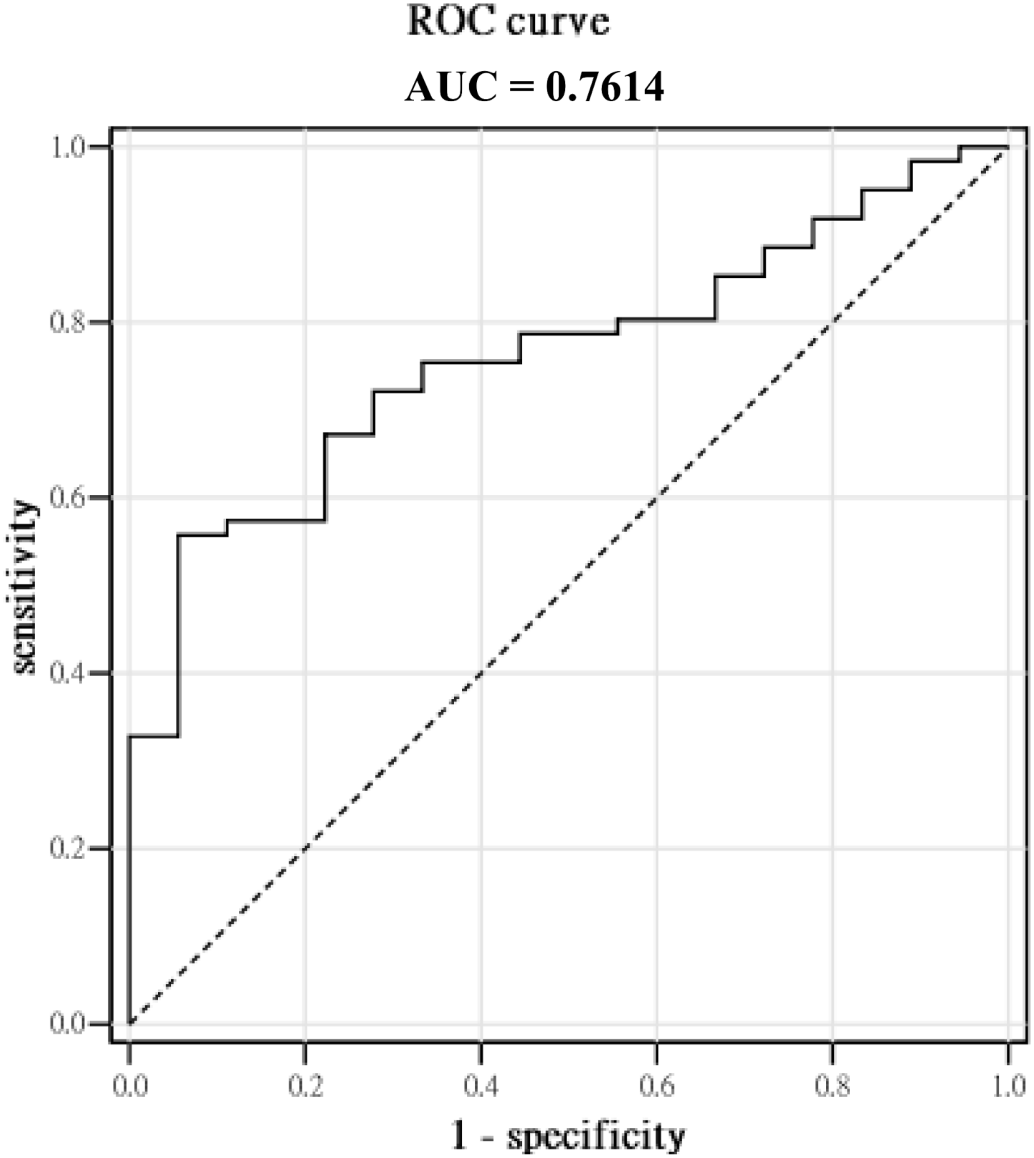
The ROC curve and measurement of AUC between decorin levels and the treatment response of novel agent-based induction.

Among the 79 patients who had received novel agent-based induction regimens, the overall response rate (ORR), in terms of partial response or better, and the depth of responses were significantly better in the H-DCN group than in the NL-DCN group (Table 3). An ROC curve was conducted and the AUC was 0.7614 (Fig 2). The estimated best cut-off for decorin level to predict treatment response was 14.57 ng/mL, which generated sensitivity and specificity of 56% and 94%, respectively. Among the 42 patients who had received conventional chemotherapeutic agent-based regimens, there were no significant differences in the ORR or depth of response between these two groups (H- and NL-DCN groups) (Table 3).

**Table 3:** The treatment response of chemotherapeutic agents-based and novel agents-based induction regimens in the H- and NL-DCN groups.

Induction (N)	Chemotherapeutic agents-based (42)			Novel agents-based (79)		
DCN group (N)	H-DCN (13)	NL-DCN (29)	*P*-value	H-DCN (33)	NL-DCN (46)	*P*-value
decorin level (ng/mL)	>15.15	< = 15.15		>15.15	< = 15.15	
		N (%)			N (%)	
CR*	0 (0)	0 (0)	0.273^#^	9 (27)	7 (15)	0.008^#^
VGPR	3 (23)	1 (3)		10 (31)	10 (22)	
PR	4 (31)	11 (38)		13 (39)	12 (26)	
SD	6 (46)	15 (52)		1 (3)	13 (28)	
PD	0 (0)	2 (7)		0 (0)	4 (9)	
ORR (> = PR)	7 (54)	12 (41)	0.516	32 (97)	29 (63)	< 0.001

*. Denote immunofixation negative complete response

^#^: Fisher’s exact test

Abbreviations: CR, complete response; DCN, decorin; VGPR, very good partial response; PR, partial response; SD, stable disease; PD, progressive disease; ORR, overall response rate

### Progression-free survival

**Fig 3:**
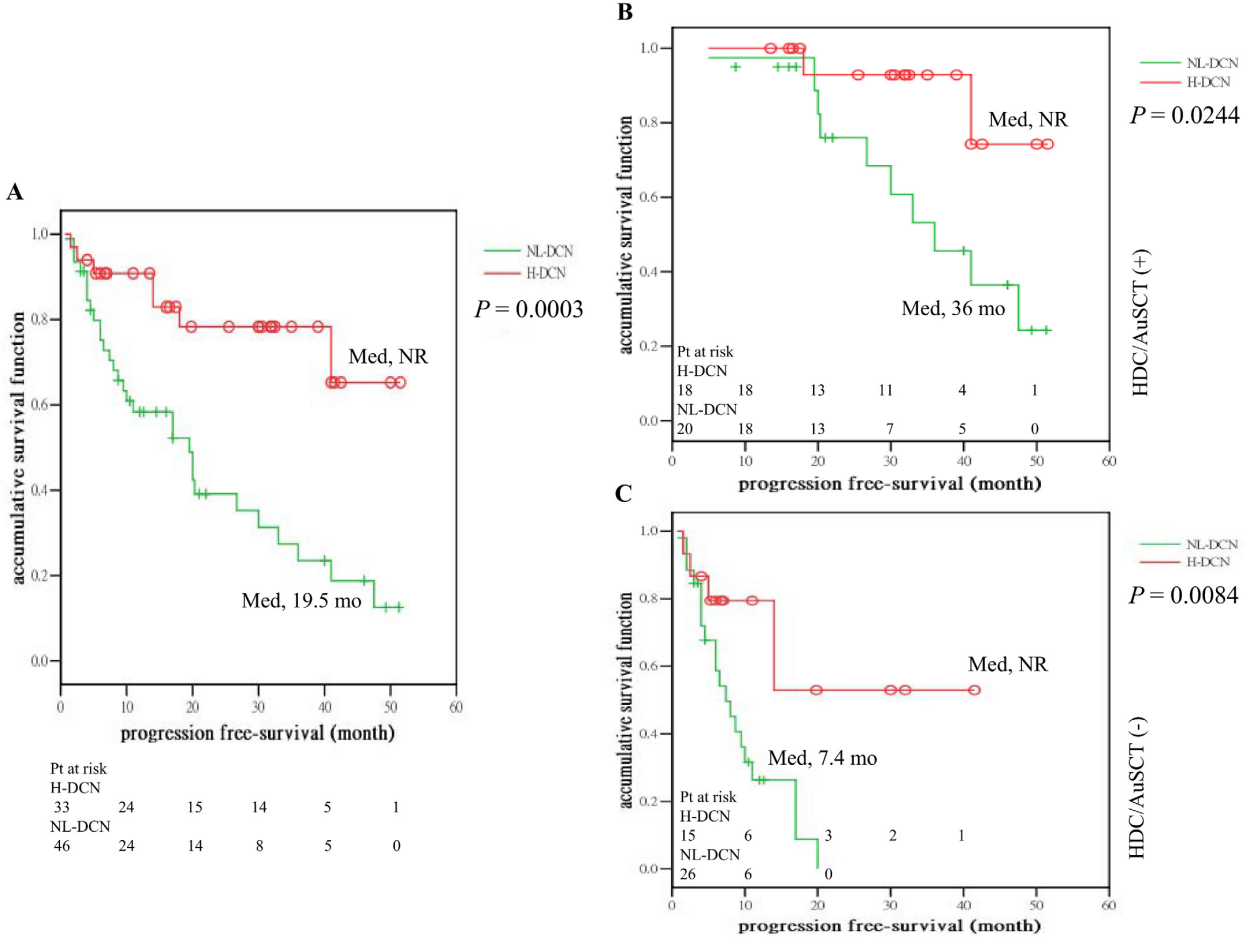
Progression-free survival (PFS) of the frontline therapy for the patients who had received novel agents-based induction regimens. A significantly longer PFS was observed in the H-DCN group compared to the NL-DCN group (A), which was observed in the patients who had further received HDC/AuSCT (B) and also in those who did not receive HDC/AuSCT (C). (Med, median; NR, not reached).

Among the 79 patients who had received novel agent-based induction regimens, after a median follow-up of 30 months (95% CI, 23.84–36.16 mo), the median PFS of frontline therapy between the H- and NL-DCN groups were significantly different (not reached vs. 19.5 mo; 95% CI, 10.57–28.43; *P* = 0.0003; Fig 3A). This difference was observed in patients who had additionally received HDC/AuSCT (n = 38; H- and NL-DCN, the median PFS, not reached vs. 36 mo; 95% CI, 24.15–47.85; *P* = 0.0244; Fig 3B), and also in those who did not receive HDC/AuSCT (n = 41; H- and NL-DCN, the median PFS, not reached vs. 7.4 mo; 95% CI, 4.36–10.44; *P* = 0.0084; Fig 3C).

### Multivariate analysis

Among the 79 patients who had received novel agent-based induction regimens, multivariate analysis confirmed H-DCN (Decorin > 15.15 ng/mL) as a significant and independent factor for treatment response (Odds ratio, 20.014 with 95% CI, 2.187–183.150; *P* = 0.008). The other independent factor associated with treatment response was C-reactive protein ≥ 0.8 mg/dL (Table 4). Cox regression analysis showed that H-DCN was an independent predictive factor for PFS (Hazard ratio, 0.135; 95% CI, 0.051–0.361; *P* < 0.001). The other independent predictive factors associated with PFS that remained significant were HDC/AuSCT and LDH (Table 5).

**Table 4:** Univariate and multivariate analysis among salient features and decorin level between patients who responded (N = 61) and who did not respond (N = 18) to the novel agents-based induction treatment.

	Without response (N = 18)	With response (N = 61)	*P*-value	Univariate analysis	Multivariate analysis
Item	N (%)			Odds ratio (95% CI)	
With EMD	5 (28)	5 (8)	0.043^#^	0.232 (0.058–0.922)*	0.226 (0.031–1.668)
Decorin level (ng/mL)			< .001		
< = 15.15	17 (94)	29 (48)		ref	ref
>15.15	1 (6)	32 (52)		18.759 (2.347–149.920)**	20.014 (2.187–183.150)**
Age > = 60 years	15 (83)	34 (56)	0.034	0.252 (0.066–0.961)*	0.253 (0.052–1.220)
CRP > = ULN (0.8 mg/dL)	12 (67)	20 (33)	0.010	0.244 (0.080–0.745)*	0.211 (0.054–0.820)*
High risk CAs	4 (22)	22 (36)	0.272	1.974 (0.578–6.741)	-

*. Statistical significance, p<0.05;

**. p<0.01

^#^: Fisher’s exact test

Abbreviations: CAs, cytogenetic abnormalities; CI, confidence interval; CRP, C-reactive protein; EMD, extramedullary disease; ULN, upper limit of normal range; ref, reference

**Table 5:** Cox regression analysis among salient clinical features associated with progression-free survival of the frontline therapy in the 79 patients with NDMM who had received novel agents-based induction.

	Univariate analysis	Multivariate analysis
Item	Hazard Ratio(95%CI)	
ISS III vs ISS I/II	2.214(1.139–4.304)*	1.396(0.674–2.893)
Decorin level (ng/mL)		
< = 15.15	ref	ref
>15.15	0.237(0.105–0.54)**	0.188(0.078–0.455)**
With vs without HDC/AuSCT	0.145(0.069–0.304)**	0.147(0.049–0.439)**
Age	1.073(1.039–1.109)**	1.016(0.972–1.061)
LDH > ULN vs < = ULN	3.412(1.636–7.113)**	2.355(1.045–5.306)**
High risk CAs	1.107(0.560–2.189)	-

*. Statistical significance, p<0.05

**. p<0.01

Abbreviations: CAs, cytogenetic abnormalities; CI, confidence interval; Cr, creatinine; HDC/AuSCT, high dose chemotherapy and autologous stem cell transplantation; ISS, International Staging System; LDH, lactate dehydrogenase; NDMM, newly diagnosed multiple myeloma; ULN, upper limit of normal range; ref, reference

### Overall survival

**Fig 4:**
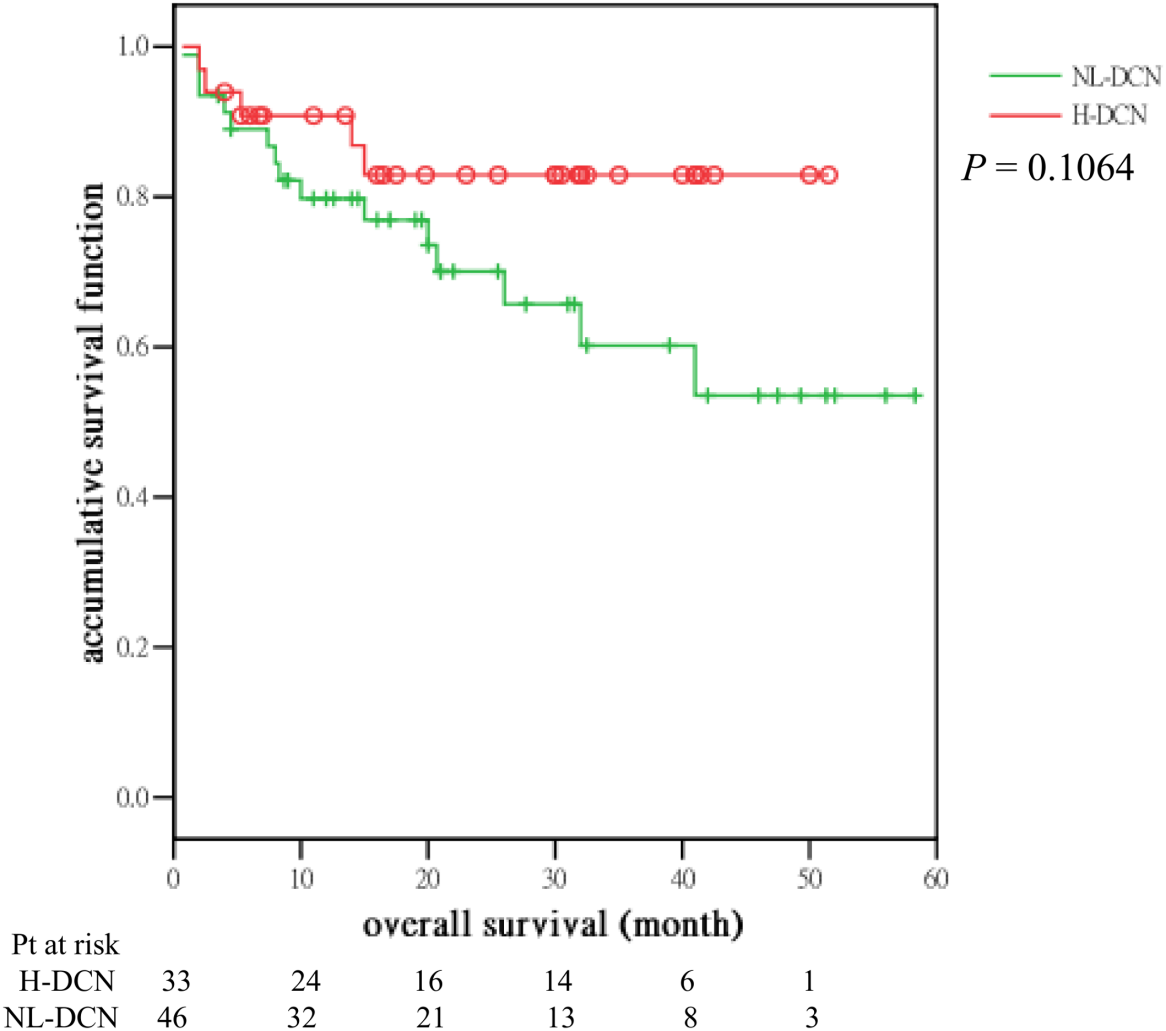
Overall survival of 79 patients with NDMM who had received novel agent-based induction therapy grouped by levels of decorin in the BM plasma at diagnosis. (H-DCN: decorin > 15.15 ng/mL; NL-DNC: decorin ≤ 15.15 ng/mL).

Until the preparation of this manuscript and among the 79 patients who had received novel agent-based induction regimens, the median OS for H- and NL-DCN groups were not reached and the differences between groups were not significantly different (*P* = 0.1064) (Fig 4).

## Discussion

To our knowledge, this is the first study to demonstrate a positive correlation between higher decorin levels in the BM plasma and superior treatment response to the novel agent-based induction regimens in patients with NDMM. Our study partly supports previous in vitro data that decorin might inhibit MCs [18, 19]. Nevertheless, the underlying mechanism remains unclear but may be attributable to several factors. First, decorin confers direct anti-myeloma effects by binding to several receptors on the MCs, such as Met, EGFR, and IGF-IR, which consequently degrades the receptors and downregulates downstream signaling, thereby inhibiting cell growth [11, 24]. Second, decorin may bind to the EGFR and IGF-IR on the vascular endothelial cells and suppress the expression of hypoxia-inducing factor-1α, thereby inhibiting angiogenesis [16, 25]. Third, higher decorin levels sequestrate increased cytokines in the BM milieu that are beneficial for the growth of MCs, such as TGF-β and IGF-I [8]. Finally, decorin could potentially augment the inhibitory power of these novel agents. PIs and IMiDs trigger the caspase-3- and caspase-8-mediated apoptosis of MCs, respectively, and inhibit BM angiogenesis [4]. IMiDs also stimulate natural killer (NK), T-, and NK-T-cells to destroy MCs [3, 4]. Similarly, decorin promotes caspase-mediated apoptosis and inhibits angiogenesis [8, 11, 16]. Intracellular accumulation of truncated decorin was found to induce ER stress [10], which may potentiate the anti-myeloma effect of PIs [3]. Particularly, the ectopic expression of decorin in human malignant glioma cells enhances the alloreactive immune response mediated by CD8^+^, CD4^+^ T cells, and NK cells [26]. To partly support this final point, the association between higher decorin level and superior treatment response was more evident in patients who had received novel agent-based inductions than in those who had chemotherapeutic agent-based inductions. Notably, there was also a trend in the MP/VAD group that patients belonged to the H-DCN group did better, terms of more deeper response and higher overall response rate, than those who in NL-DCN group, although the significance level was not reached due to possibly the limited patient numbers (Table 3). Therefore, although the anti-myeloma effect of decorin could be (synergistically) enhanced by novel-agent based treatment, a similar effect between decorin and the chemotherapeutic agents could not be totally excluded. Actually, a synergism between decorin and a chemotherapeutic agent, carboplatin has been reported [27]. The possible synergism between decorin and those novel agents targeted BM is still a hypothesis, and need further laboratory work to confirm. Nonetheless, this observation may provide a basis for further mechanistic investigation on decorin in the BM stroma of patients with MM, along with other novel agents, to purge the tumor niche and thereby inhibit growth of MCs.

Our study also showed that higher decorin levels were associated with longer PFS regardless of whether induction was followed by HDC/AuSCT and post-AuSCT maintenance (Fig 3), supporting previous in vitro evidence that decorin might directly or indirectly inhibit growth of MCs [18]. This effect seemed to be independent of other prognostic factors. The other factors that remained independent were HDC/AuSCT and LDH, which are known important factors associated with PFS in MM patients [28]. Another important prognostic factor is the high-risk cytogenetics, which was not significantly associated with PFS in our patients (Tables 4 and 5). The reason for this non-association may be that the novel combination of BTD and BTD+Cy is able to partly overcome the poor outcome from these high risk cytogenetics, such as t (4;14) and del (17p) [29]. The reason why age for the H-DCN group was younger than the NL-DCN group is unclear, but which might indeed contribute partly to the better outcome seen in the H-DCN-group, e.g. younger patients may have better general condition, and better immunity etc.

The molecular mechanism by which decorin expression is switched on or off is unclear. Epigenetic control, including hypermethylation of the promoter region of decorin, might play a role in silencing the decorin gene [8]. Very recently, chemokine ligand 3 was found to downregulate decorin expression [30], as was the use of aminobisphosphonates [31]. Although decorin may sequester TGF-β in the ECM, it was recently reported that TGF-β might induce decorin downregulation through micro RNA-181b [32]. By contrast, decorin has been reported to be upregulated by the parathyroid hormone and certain demethylating agents [33]. Intriguingly, the PIs (e.g., BTZ, CFZ) have been demonstrated to increase decorin expression in OBs [18]. Additional studies are needed to determine whether increased decorin levels within the BM, along with novel agents that target the BM stroma, would be a more effective strategy for induction remission and long-term disease control in MM patients. To this point, it is worth considering how to define a “higher” or clinically relevant level of decorin. In our study, it seemed that the H-DCN (decorin > 15.15 ng/mL) group showed significantly longer PFS than the NL-DCN group. The level was set just above the highest level of decorin from our 22 normal BM donors (15.14 ng/mL), which was approximately the median in addition to 2 SD. Meanwhile, the approximate AUC of 0.8 from the ROC curve suggested acceptable to excellent discrimination for decorin to predict the treatment response of novel agent-based induction [34], and by this modeling, the best cut-off for decorin level was 14.57 ng/mL, which is very close to 15.15 ng/mL. Therefore, the “higher” level of decorin, although not yet clearly defined, might convey some biological and clinical significance in MM and is worth being validated in future studies.

Notably, the median decorin level in the BM plasma of our normal control (n = 22; median, 10.31 ng/mL) was much lower than the report level from the Kristensen et al (n = 23; median, 35.2 ng/mL) [16], although the sample size of these normal controls and the detection kit used for decorin level were generally the same. The reason for this difference is unclear. All the plasma samples in our study were analyzed at least twice, and the mean intra-assay CV was 4%, which was comparable to or slightly better than that (8%) reported from the Kristensen et al [16]. There was a very wide range of decorin levels for the healthy volunteers in that report and unfortunately the details for their healthy volunteers were not available [16], but all of our normal controls were healthy BM donors. Intriguingly, we found a positive correlation between decorin level and age in our normal BM donors, suggesting decorin level might be variable among different age groups. Notably, the median age of our normal control was lower than that reported by the Kristensen et al [16] (46 vs. 59 years, respectively). Archived samples might also be a problem, but the decorin levels between those samples collected before and after 2010 were not different in this study. Because the decorin levels in BM plasma between different ethic populations have never been reported, the possibility that these differences resulted from different populations cannot be completely excluded. Moreover, different from Todoerti et al [17], the level of decorin in our study did not correlate with bone lesions (Table 1). This finding that decorin level was not related to lytic bone lesions in patients with MM was similar to another report [16]. To support this idea, mice with targeted disruption of the biglycan gene, but not the decorin gene, have impaired postnatal bone formation [9, 35]. Notably, Todoerti et al [17] analyzed the transcriptional expression levels of decorin from BMMSCs and OBs rather than the translational level of decorin in the whole BM milieu, as in our study.

This study had several limitations. First, because of the retrospective nature of this study, variations among individual patients resulted in biased response and outcome assessments. However, the treatment responses among these various induction regimens in our patients were comparable to other studies [29, 36]. Compatible with other studies [36], adding cyclophosphamide to BTD (BTD+Cy) did not provide significantly additional activity compared to BTD. Therefore, these two regimens could be considered similar. Second, decorin is just one of the complex factors that affect the ECM of BM; interactions among the other ECM components should be elucidated in future studies. However, a recent study indicated that the expression of only decorin, but not the other related SLPRs such as asporin and biglycan, varied between tumor and normal tissues [37]. Moreover, decorin, but not biglycan, could inhibit the metastasis of the targeted tumor cells through transgenic delivery [38]. These data suggest a unique role of decorin in cancer-related ECM.

### Conclusions

Higher decorin levels in the ECM of the BM were associated with superior treatment response and outcome to novel agent-based induction regimens as part of frontline therapy in patients with NDMM. These findings disclose the potential role of decorin in MM and provide a basis for further study on possible synergistic anti-myeloma effects between decorin and the novel agents that target BM stroma.

## Supporting Information

S1 Fig The disposition and median treatment duration, as well as cycles of the various induction regimens, among the 121 patients with NDMM. (PPTX)

S1 Table The comparison on induction regimens between the H- and the NL-DCN groups. (DOCX)

S2 Table Raw data for normal control. (XLSX)
