# Supplementary material for: Higher Decorin Levels in Bone Marrow Plasma Are Associated with Superior Treatment Response to Novel Agent-Based Induction in Patients with Newly Diagnosed Myeloma - A Retrospective Study
Source: PLoS One. 2015 Sep 17;10(9):e0137552. doi: 10.1371/journal.pone.0137552 (PMC4574783; doi:10.1371/journal.pone.0137552)
Supplement: S1 Fig — (PPTX) [file pone.0137552.s001.pptx]

## Slide 1
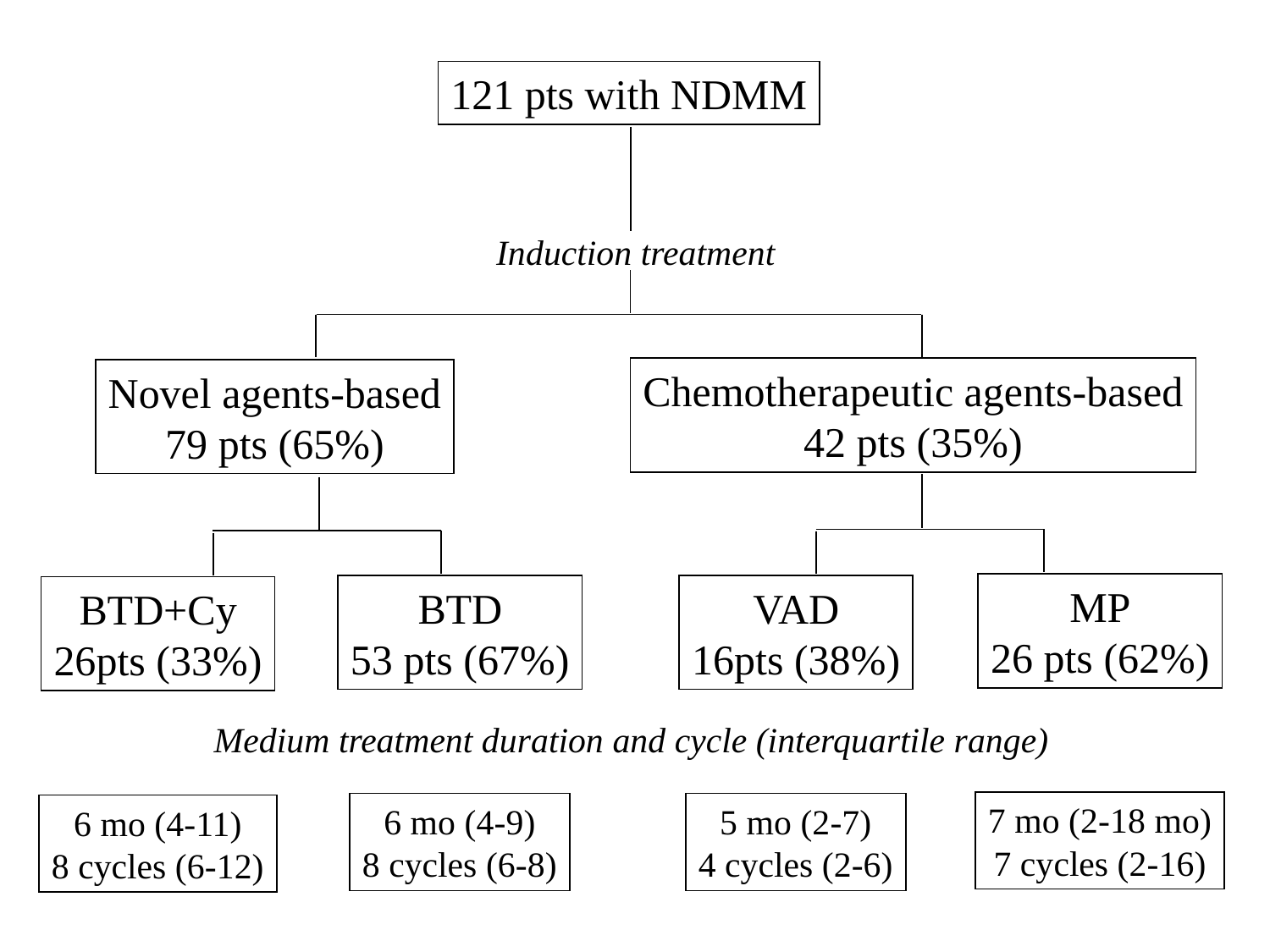

121 pts with NDMM
Induction treatment
Chemotherapeutic agents-based
42 pts (35%)
Novel agents-based
79 pts (65%)
MP
26 pts (62%)
VAD
16pts (38%)
BTD
53 pts (67%)
BTD+Cy
26pts (33%)
Medium treatment duration and cycle (interquartile range)
7 mo (2-18 mo)
7 cycles (2-16)
5 mo (2-7)
4 cycles (2-6)
6 mo (4-9)
8 cycles (6-8)
6 mo (4-11)
8 cycles (6-12)
