# Supplementary material for: Higher Decorin Levels in Bone Marrow Plasma Are Associated with Superior Treatment Response to Novel Agent-Based Induction in Patients with Newly Diagnosed Myeloma - A Retrospective Study
Source: PLoS One. 2015 Sep 17;10(9):e0137552. doi: 10.1371/journal.pone.0137552 (PMC4574783; doi:10.1371/journal.pone.0137552)
Supplement: S1 Table — (DOCX) [file pone.0137552.s002.docx]

**S1 Table. The comparison on induction regimens between the H- and the NL-DCN groups**

|  | H-DCN  (N=46) | NL-DCN  (N=75) |  |
| --- | --- | --- | --- |
| Induction regimen | N (%) | | *P*-value |
|  |  | | 0.241 |
| Chemotherapeutic agents-based  VAD  MP | 6 (13)  7 (15) | 10 (13)  19 (25) |  |
| Novel agents-based  BTD  BTD+Cy | 19 (41)  14 (31) | 34 (45)  12 (17) |  |
